# *N*-Carbamoylglutamate Supplementation on the Digestibility, Rumen Fermentation, Milk Quality, Antioxidant Parameters, and Metabolites of Jersey Cattle in High-Altitude Areas

**DOI:** 10.3389/fvets.2022.848912

**Published:** 2022-04-04

**Authors:** Zixin Liu, Fuyong Yan, Hui Mi, Xiaokang Lv, Kaijun Wang, Bin Li, Tao Jin, Liang Chen, Guijie Zhang, Ximei Huang, Chuanshe Zhou, Zhiliang Tan

**Affiliations:** ^1^CAS Key Laboratory for Agri-Ecological Processes in Subtropical Region, National Engineering Laboratory for Pollution CON and Waste Utilization in Livestock and Poultry Production, Hunan Provincial Key Laboratory of Animal Nutrition Physiology and Metabolic Process, Institute of Subtropical Agriculture, Chinese Academy of Sciences, Changsha, China; ^2^University of Chinese Academy of Sciences, Beijing, China; ^3^Hunan Jiuding Technology (Group) Co., Ltd, Changsha, China; ^4^College of Animal Science and Technology, Guangxi University, Nanning, China; ^5^Institute of Animal Science of Tibet Academy of Agricultural and Animal Husbandry Sciences, Lhasa, China; ^6^School of Agriculture, Ningxia University, Yinchuan, China; ^7^Changsha Green Top Biotech Co., Ltd, Changsha, China

**Keywords:** Jersey dairy cows, NCG supplements, metabolism, high altitude, milk quality, feed additives, digestibility, rumen fermentation

## Abstract

This study aimed to assess the impact of the dietary supplementation of *N-*carbamoylglutamate (NCG) on nutrient digestibility, rumen fermentation, milk quality, oxidative stress, and metabolites in the plasma and feces of Jersey cattle under high altitude with the hypoxic condition. A total of 14 healthy lactating Jersey dairy cows with similar body conditions were selected and randomly divided into 2 groups. The control group (CON group, *N* = 6 replicates) was fed with a conventional complete diet, whereas the experimental group (NCG group, *N* = 8 replicates) received 20 g/d per head NCG supplementation. The experiment lasted for 60 days, the adaptation period was 12 days, and the formal experiment period was 48 days. Except that the NCG group showed an upward trend in dry matter intake (DMI) (*p* = 0.09) and the fermentation parameters, the molar proportion of butyric acid tended to decrease (*p* = 0.08); the two groups had no significant differences (*p* > 0.05) in nutrients digestibility, plasma immunity, and antioxidant ability. However, compared with the CON group, the milk fat rate and blood oxygen saturation of the NCG group showed an upward trend (*p* = 0.09). For indexes associated with altitude stress, the contents of thyroxine, transferrin, and endothelin both decreased significantly (*p* < 0.05) in the NCG group. Meanwhile, heat shock protein (*p* = 0.07) and aldosterone (*p* = 0.06) also showed a downward trend. A total of 114 different metabolites were identified from feces and plasma, 42 metabolites were derived from plasma that mainly included 5 kinds of Super Class, and 72 metabolites were derived from feces that mainly included 9 kinds of Super Class. The significantly increased plasma differential metabolites were 2,5-dihydroxybenzoate and salicyluric acid, and the significantly increased fecal differential metabolites were Butenafine (fold change > 2). Pathway analysis showed that after applying NCG as a feed additive, the changes of the Jersey dairy cows mainly focused on amino acid metabolism and lipid metabolism. These results indicated that adding NCG to the diet can prevent the hypoxic stress state of lactating Jersey cows in high-altitude areas and has a tendency to improve milk quality.

## Introduction

The environment of Tibet is low-pressure and hypoxic due to the high average altitude (average altitude of 4,000 m) (1), and animals in this region are prone to a series of pathophysiological changes caused by high-altitude hypoxia (HAH). Metabolic dysfunction and the occurrence of various clinical symptoms are the main problems for high-altitude animals (2–5). Generally, HAH causes an increase in the prevalence of high-altitude pulmonary hypertension (HAPH) in cattle (6), an increase in intestinal permeability (7), a decline in milk production (8), and many other problems that have severely affected the physical functions of animals, resulting in low production capacity, which deeply troubled and restricted the development of animal husbandry in this region.

The Jersey cattle, which originated in the United Kingdom, has been widely introduced in various regions including Tibet due to its strong disease resistance, rough feed tolerance, high feed utilization rate, and high milk fat content (that as the origin of butter and it is life necessity for local people). The milk production of Jersey cattle is the second behind the Holstein cow among scalper breeds, and crossing with other breeds of cattle can significantly increase the milk fat rate of offspring (9). Because of the early sexual maturity and the high conception rate of the Jersey cattle, the production performance of the offspring produced by the crosses between the Jersey cattle and the local cattle in Tibet has been significantly improved. It is mainly manifested in many aspects such as the increase in birth weight, growth rate, and the increase in milk production (10). However, at the same time, long-term exposure to a low-oxygen environment has caused the production performance of the Jersey cattle to still lag behind that of the plains and cannot reach the optimal level. This problem still needs to be resolved (11).

The use of feed additives is one of the common ways to improve animal production performance. As a typical nutritional feed additive, arginine (Arg) can effectively improve the immune function and production performance of dairy cows (12, 13), but it is limited due to its high cost of addition and antagonism reaction with other amino acids. At this time, *N*-carbamoylglutamate (NCG) can promote the synthesis of endogenous Arg and has become a suitable alternative. NCG is a structural analog of *N*-acetylglutaminase (NAG), that is an allosteric activator of carbamoyl phosphate synthase-I (CPS-I), and CPS-I is the rate-limiting enzyme for the endogenous synthesis of Arg. At the same time, NAG can also activate dihydropyrrole-5-carboxylic acid synthase to promote the synthesis of glutamine or proline citrulline, thus promoting the generation of Arg (14). Arg has many biological functions such as regulating vascular tension, blood flow, and blood pressure (15), and studies have shown that the application of Arg at high altitude can increase blood oxygen saturation and improve the symptoms of altitude sickness such as HAH and HAPH caused by hypoxia and other factors (16). At the same time, because NAG is unstable and easily hydrolyzed into acyl amino acids under the action of acyl decomposing enzymes, and Arg is easily degraded in the rumen of ruminants, NCG has become a potential substitute for Arg (17, 18). Through the safety and toxicological experiments on a large number of rats and human volunteers, it has been proved that an appropriate amount of NCG has no toxic side effects on humans and animals (19, 20). At present, there have been relatively few reports on the effects of NCG on ruminants compared to more NCG addition studies focusing on mice and pigs. In existing reports, it was found that supplementation of NCG tends to increase milk production (*p* = 0.07) and supplementation with 20 (g/d/head) has the highest milk yield and the lowest concentration of urea nitrogen that could be recycled by ornithine–urea (21, 22). Since the existing reports on NCG are all effective in low-altitude areas, faced with the geographical differences between low-altitude and high-altitude areas, there is still no research on the alleviating effect of NCG supplement on HAH in dairy cows. Therefore, whether the supplementation of NCG in feed can also affect the physiological state and production performance of dairy cows is a question worth exploring.

This study describes the results of applying NCG as a feed additive to Jersey cattle in the plateau region, to better understand the relationship between the physiological state and the production performance of dairy cows under hypoxic condition in the high-altitude regions, to provide the theoretical basis and data support for promoting the development of animal husbandry in high-altitude areas.

## Materials and Methods

### Animal Management and Experimental Design

In Qunipa, Dazi District, Lhasa City, Tibet Autonomous Region (Latitude N: 29°48′22.54″, Longitude E: 91°37′14.89″, Altitude: 3,700 m), 16 Jersey cattle with an average body weight (BW) of 385.36 ± 46.25 kg (mean ± SD) were used as experimental animals. The experiment was carried out in a completely random design. All Jersey cattle were randomly assigned into two groups, with 8 animals in each group, the control group (CON group) were feeding basis total mixed rations (TMR) and the experimental group were feeding basis TMR plus 20(g/d/head) *N*-carbamylglutamate (NCG), the ingredients and chemical composition of the diets are given in Table 1. Jersey cattle were fed two equal meals at 08.00 and 18.00 h daily. All animals had free access to fresh water. The total experimental period lasted for 60 days, the acclimatization period lasted for 12 days until all Jersey cattle reached the stable dry matter (DM) intake according to the standard of metabolic BW, and the formal experimental period lasted for 48 days. The experimental feeds were offered *ad libitum* and the refusals were collected every day during the formal experimental period to measure the voluntary feed intake, and feed intake was measured.

**Table 1:** Ingredients and chemical composition of the experimental diet.

**Items**	**Content**
**Ingredients**	
Hulless barley straw (%)	35
Corn silage (%)	25
Corn (%)	17.15
Wheat bran (%)	1.8
Soybean meal (%)	8.9
Rapeseed cake (%)	8
Ca(HCO3)_2_ (%)	0.15
Fat powders (%)	2.4
NaCl (%)	0.6
Premix^a^ (%)	1
Total (%)	100
**Nutrient levels^b^**	
GE (MJ/kg)	17.90
CP (%)	8.84
NDF (%)	62.41
ADF (%)	36.78
Ca (%)	0.85
P (%)	0.51

a *The premix provided the following per kg of diets:50-g Mg, 2.5-g Fe, 0.4-g Cu, 2 g Mn, 1.5-g Zn, 10-mg Se, 25-mg I, 5-mg Co, 80,000 IU vitamin A, 12,500 IU vitamin D, and 1,250-mg vitamin E*.

b *GE, gross energy; CP, crude protein; NDF, neutral detergent fiber; ADF, acid detergent fiber; Ca, calcium; P, phosphorus*.

### Sample Collection and Handling

During the experiment, 2 cows in the CON group developed physiological diseases, and the final samples were 6 cows in the CON group and 8 cows in the experimental group. The diets offered and left behind were recorded daily for calculating DMI and collected residual feed samples were used for chemical analysis. Feed samples were oven-dried at 65°C, ground to pass a 1-mm sieve, and stored for pending laboratory analysis. On day 41 of the formal experiment, before morning feeding, 5 ml blood sample was collected from each cattle in a vacuum tube pre-filled with Ethylene diamine tetraacetic acid (EDTA) for metabolomic assays, and an additional 5 ml blood sample was collected from each cattle in a vacuum tube pre-filled with heparin for blood parameters determinations. After 30 min still standing, samples were centrifuged at 3,000 rpm, 1 ml plasma was taken in the tube and immediately frozen by liquid nitrogen, and 3 replicates tubes of each cattle were stored at −80°C for blood parameters, antioxidant capacity, immunity, parameters associated with altitude sickness determining, and Liquid chromatography tandem-mass spectrometry (LC–MS/MS) analysis. On day 41 before morning feeding, fecal samples were collected in sterile plastic tubes, immediately frozen in liquid nitrogen, and stored at −80°C until mixed up for LC–MS/MS analysis. On day 42, 43, 44, 45, and 46 of the formal experiment, at 0, 3, and 6 h after morning feeding, 50-g fresh feces were taken from the rectum for nutrients determination except for crude protein (CP), another 50-g fresh feces were taken from rectum and added 10 ml 10% H_2_SO_4_ for CP determination, the feces samples in each day were mixed up and oven dried at 65°C, then ground to pass a 1-mm sieve for nutrients determination. On day 47 and 48 of the formal experiment, rumen contents were collected orally using an oral stomach tube at 0, 3 and 6 h after morning feeding, the first 100 ml of rumen contents were discarded, and the following 100 ml were rapidly collected for sampling (23) and stored at −80°C. After mixing the rumen contents from the 3 time points, approximately 10 ml of the contents was centrifuged at 10,000*g* for 15 min at 4°C, 1 ml supernatant was transferred into tubes containing 0.1 ml of 25% (w/v) metaphosphoric acid, and this mixture was stored at −20°C for subsequent determination of volatile fatty acids (VFAs) and ammonia. On day 47 and 48 of the formal experiment, 50 ml milk samples were collected at 6.00 and 19.00 h each day, The potassium dichromate preservative was added to the milk and mixed with the four milk samples in equal proportion. Composited milk samples were stored at 4°C and analyzed for milk quality determination.

### Samples Analysis

Feeds and feces were used for nutrients determination, the gross energy (GE) was determined by an isothermal automatic calorimeter (5E-AC8018, Changsha Kaiyuan Instruments Co., Ltd, China). The DM, CP, GE, organic matter (OM), calcium (Ca), and phosphorus (P) were determined according to Association of Official Analytical Chemists methodologies (AOAC, 2002). Neutral detergent fiber (NDF) and acid detergent fiber (ADF) were determined using a Fibretherm Fiber Analyzer (Gerhardt, Bonn, Germany) according to Van Soest et al. (24). Apparent total tract digestibility (ATTD) was calculated using acid insoluble ash (AIA) endogenous indicator method with the following formula (25):


(1) $$\begin{array}{l} ATTD\;(\%)\;=\;100\;\times \;\{1\;-\;[\;(fecal\;DM\;nutrient\;\%)\\ \;\times (feed\;DM\;AIA\;\%)]\;/\;[(feed\;DM\;nutrient\;\%)\\ \;\times \;(fecal\;DM\;AIA\;\%)\;]\}. \end{array}$$

The NH_3_-N and VFA concentrations were determined according to Chen et al. (26). Milk samples were measured using Basic Unit MilkoScan FT + Type 76150 (Foss Electric, Hillerd, Denmark), blood biochemical parameters and immunity parameters (IgA, IgG, and IgM) were determined by Shenzhen Mindray BS-190 fully automatic biochemical analyzer (Shenzhen Mindray Bio-Medical Electronics Co., Ltd, Shenzhen, China). Concentration of antioxidant parameters (superoxidase dismutase, catalase, malondialdehyde (MDA), total antioxidant capacity (T-AOC) in plasma were analyzed by kits (Nanjing Jiancheng Bioengineering Institute Co., Ltd, Nanjing, China). Parameters associated with altitude sickness in plasma were determined by ELISA kits (Nanjing Jiancheng Bioengineering Institute Co., Ltd, Nanjing, China). Ammonia nitrogen (NH_3_-N) and VFA were measured by the methods of 22. Blood oxygen saturation and heart rate were detected by pulse oximeter (CMS60D-VET, Qinhuangdao CONTEC Instruments Co., Ltd, China).

### Metabolite LC–MS/MS Analysis

The LC–MS/MS analyses of plasma and feces were performed using an Ultra High Performance Liquid Chromatography (UHPLC) system (1290, Agilent Technologies) with a UPLC HSS T3 column (2.1 mm × 100 mm, 1.8 μm) coupled to Q Exactive (Orbitrap MS, Thermo). The mobile phase A was 0.1% formic acid in water for positive, and 5 mmol/L ammonium acetate in water for negative, and the mobile phase B was acetonitrile. The elution gradient was set as follows: 0 min, 1% B; 1 min, 1% B; 8 min, 99% B; 10 min, 99% B; 10.1 min, 1% B; 12 min, 1% B. The flow rate was 0.5 ml/min. The injection volume was 2 μl. The Q Exactive (QE) mass spectrometer was used for its ability to acquire Tandem mass spectrometry (MS/MS) spectra on an information-dependent basis (IDA) during an Liquid chromatography coupled with mass spectrometry (LC/MS) experiment. In this mode, the acquisition software (Xcalibur 4.0.27, Thermo) continuously evaluates the full scan survey MS data as it collects and triggers the acquisition of MS/MS spectra depending on preselected criteria. Electrospray ionisation (ESI) source conditions were set as following: sheath gas flow rate as 45 Arb, Aux gas flow rate as 15 Arb, capillary temperature as 320°C, full MS resolution as 70,000, MS/MS resolution as 17,500, collision energy as 20/40/60 eV in Normalized collision energy (NCE) model, spray voltage as 3.8 kV (positive) or −3.1 kV (negative), respectively.

### Metabolomics Data Preprocessing and Analysis of Metabolomics

All the MS raw data (.raw) files were converted to the mzML format using ProteoWizard, and processed by R package XCMS (version 3.2). The preprocessing results generated a data matrix that consisted of the retention time (RT), mass-to-charge ratio (m/z) values, and peak intensity. OSI-SMMS (version 1.0, Dalian Chem Data Solution Information Technology Co. Ltd) was used for peak annotation after XCMS data processing with in-house MS/MS database. After obtaining the sorted data, Principal Component Analysis (PCA) was performed and transformed into linear uncorrelated variables through orthogonal transformation, and a low-dimensional projection in PCA was formed. The plasma and feces samples of the NCG group and the CON group were analyzed based on LC–MS technology, using SIMCA software (V14.1, Sartorius Stedim Data Analytics AB, Umea, Sweden) to perform logarithmic conversion and centralization of the data dPCA was performed after formatting. To avoid the difference, variable was scattered to more principal components, the data is further used orthogonal partial least squares–discriminant analysis (OPLS–DA). The corresponding OPLS–DA model to obtain the *R*^2^ value and *Q*^2^ value of the random model was established to avoid the over-fitting of the test model and to evaluate the statistical significance of the model. The standard for screening differential metabolites was Student's t-test *p* < 0.05, and variable importance in the projection (VIP) was calculated in OPLS–DA model greater than 1. By searching the chemical structure of these differential metabolites in the Human Metabolome Database (HMDB), Kyoto Encyclopedia of Genes and Genomes pathway database (KEGG) and other published articles, and classifying them on the Super Class and Class levels based on the HMDB classification criteria. Differential metabolites between 2 groups mapped into their biochemical pathways through metabolic enrichment and pathway analysis based on MetaboAnalyst 5.0 (https://dev.metaboanalyst.ca).

### Statistical Analysis

The data of the nutrients digestibility, rumen fermentation, milk quality, and antioxidant parameters and index associated with altitude stress of the two groups were analyzed using the Shapiro–Wilk test for normal distribution with subsequent Student's t-test to compare means in the SPSS Statistics 22 (IBM, Chicago, USA) software. A *p* ≤ 0.05 was considered to indicate a statistically significant difference, and 0.05 < *p* < 0.10 represented a tendency.

## Results

### Nutrients Digestibility

The nutrients digestibility parameters were presented in Table 2, we found that feed NCG to Jersey dairy cows was tended to increase DMI (*p* = 0.09) and the digestibility of DM, CP, NDF, ADF, GE, and OM were not altered (*p* > 0.1) by supplementation of NCG.

**Table 2:** Effect of NCG on nutrients digestibility.

**Items^a^**	**Treatments^b^**		**SEM^c^**	***P***
	**CON**	**NCG**		
DMI, kg/d	10.57	10.94	0.19	0.09
**Apparent digestibility**				
DM (%)	64.36	64.09	2.95	0.26
CP (%)	76.36	72.73	1.71	0.31
NDF (%)	66.49	62.36	1.46	0.17
ADF (%)	57.93	58.31	1.97	0.93
GE (%)	65.30	64.37	2.96	0.76
OM (%)	65.85	65.37	0.29	0.12

a *DMI, dry matter intake; DM, dry matter; CP, crude protein; NDF, neutral detergent fiber; ADF, acid detergent fiber; GE, gross energy; OM, organic matter*.

b *CON, CON group, N = 6, basal diet without supplementation of N-carbamylglutamate; NCG, N = 8, treatment that added 20 (g/d/head) N-carbamylglutamate*.

c *SEM was standard error of means*.

### Milk Quality

As shown in Table 3, the milk fat content was tended to increase by 84.96% (*p* = 0.09), and the milk protein content in NCG group (5.09%) was also tend to decrease (*p* = 0.07) when compared with the CON group (6.47%). However, lactose content (*p* = 0.74), DM content (*p* = 0.31), DM without fat (*p* = 0.24), and somatic cells (*p* = 0.30) had no significant difference between NCG group and CON group.

**Table 3:** Effect of NCG on milk quality from experimental Jersey cattle.

**Items**	**Treatments^a^**		**SEM^b^**	***P***
	**CON**	**NCG**		
Somatic cell count (×1,000 cells/ml)	4,293	3,177	516	0.30
Milk fat (%)	2.26	4.18	0.56	0.09
Milk protein (%)	6.47	5.09	0.38	0.07
Lactose (%)	1.26	1.10	0.22	0.74
Dry matter (%)	10.29	11.78	0.69	0.31
Dry matter without fat (%)	8.41	7.65	0.30	0.24

a *CON, CON group, N = 6, basal diet without supplementation of N-carbamylglutamate; NCG, N = 8, treatment that added 20 (g/d/head) N-carbamylglutamate*.

b *SEM was standard error of means*.

### The Rumen Fermentation Parameters

Rumen fermentation parameters including ammonia nitrogen (NH_3_-N) and VFAs were summarized in Table 4. The NCG supplementation had no effect (*p* = 0.44) on the NH_3_-N concentration in the rumen. The molar content of total VFA and acetic/propionic were similar (*p* > 0.10) between the treatments. For individual VFA, except the molar proportion of butyric acid was tended to decrease (*p* = 0.08) in NCG group, other VFA like the molar proportion of acetic acid, propionic acid, butyric acid, valeric acid were also similar (*p* > 0.10) between NCG and CON group.

**Table 4:** Effect of NCG on rumen fermentation parameters of experimental Jersey cattle.

**Items**	**Treatments^a^**		**SEM^b^**	***P***
	**CON**	**NCG**		
NH_3_-N (mg/dl)	9.72	10.67	0.57	0.44
Acetic acid (mol/100 mol)	66.13	66.45	0.72	0.65
Propionic acid (mol/100 mol)	18.58	17.25	1.19	0.29
Isobutyric acid (mol/100 mol)	0.63	0.64	0.07	0.85
Butyric acid (mol/100 mol)	11.68	10.18	0.78	0.08
Valeric acid (mol/100 mol)	0.55	0.60	0.10	0.63
Isovaleric acid (mol/100 mol)	1.17	1.19	0.09	0.87
Total VFA (mM)	73.42	72.11	1.72	0.73
Acetic/Propionic	3.44	3.44	0.07	1.00

a *CON, CON group, N = 6, basal diet without supplementation of N-carbamylglutamate; NCG, N = 8, treatment that added 20 (g/d/head) N-carbamylglutamate*.

b *SEM was standard error of means*.

### The Oxygen Saturation and Heart Rate

**Figure 1:**
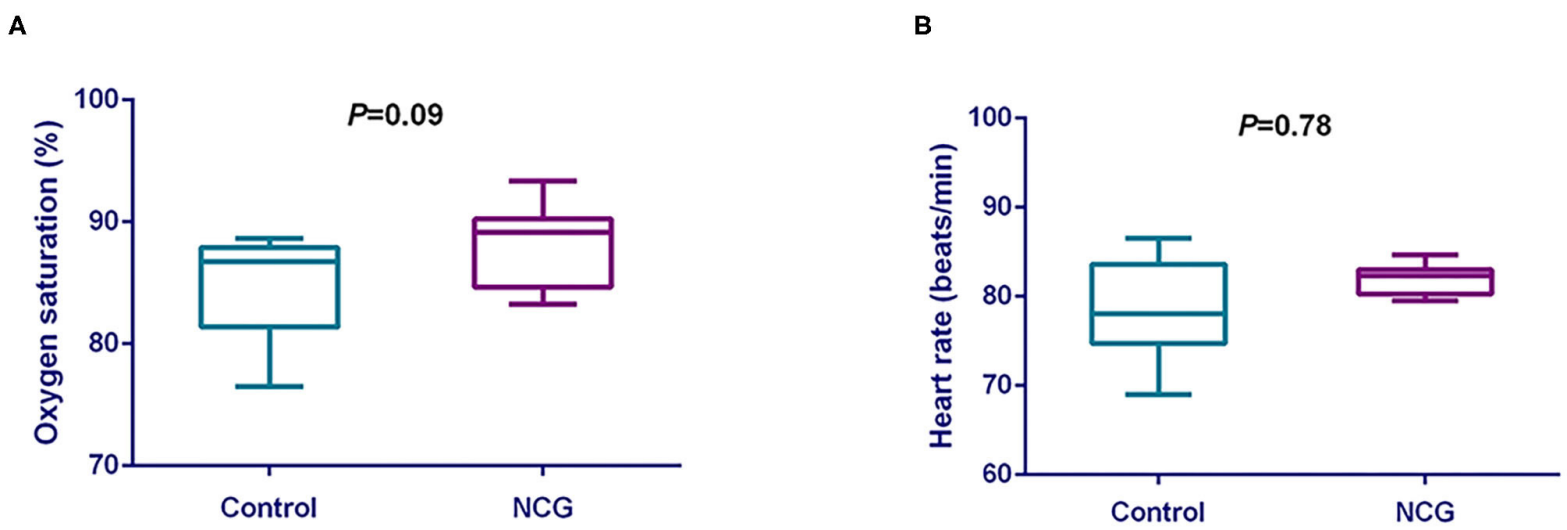
**(A)** Comparative analysis of the effect of NCG on oxygen saturation of experimental Jersey cattle. **(B)** Comparative analysis of the effect of NCG on heart rate of experimental Jersey cattle. The blue box represents the diet without NCG supplementation (Control, CON group, *N* = 6), and the purple box represents the experimental group with 20 (g/d/head) NCG added to the diet (NCG, NCG group, *N* = 8).

The oxygen saturation and heart rate were shown in Figure 1. The oxygen saturation (Figure 1A) was tended to increase (*p* = 0.09) in NCG group when compared with CON group. However, the heart rate (Figure 1B) was not different (*p* = 0.78) between treatments.

### The Plasma Immunity, Antioxidant, and Biochemical Parameters

The plasma immunity and antioxidant parameters are shown in Table 5. Immunoglobulin A (IgA), immunoglobulin G (IgG), and immunoglobulin M (IgM) concentrations in plasma did not differ (*p* > 0.10) between treatments. In terms of antioxidant parameters, such as nitric oxide synthase (NOS), superoxide dismutase (SOD), MDA, catalase (CAT) and T-AOC were similar (*p* > 0.10) between treatments.

**Table 5:** Effect of NCG on plasma immunity and antioxidant parameters of experimental Jersey cattle.

**Items^a^**	**Treatments^b^**	**SEM^c^**	***P***	
	**CON**	**NCG**		
**Immunity**				
IgA (μg/ml)	589	603	13.61	0.63
IgG (μg/ml)	4,427	4,174	152.04	0.43
IgM (μg/ml)	1,432	1,346	58.50	0.49
**Antioxidant ability**				
NOS (U/ ml)	3.19	2.97	0.19	0.59
SOD (U/ ml)	124.18	122.45	2.42	0.73
MDA (nmol/ml)	5.71	4.41	0.70	0.38
CAT (U/ ml)	0.05	0.05	0.00	0.33
T-AOC (mmol/L)	26.95	26.27	0.30	0.27

a *IgA, immunoglobulin A; IgG, immunoglobulin G; IgM, immunoglobulin M; NOS, nitric oxide synthase; SOD, superoxide dismutase; MDA, malondialdehyde; CAT, Catalase; T-AOC, total antioxidant capacity*.

b *CON, CON group, N = 6, basal diet without supplementation of N-carbamylglutamate; NCG, N = 8, treatment that added 20 (g/d/head) N-carbamylglutamate*.

c *SEM was standard error of means*.

The results of index associated with altitude stress are shown in Table 6. Compared with the CON group, the plasma contents of transferrin, thyroxine, and endothelin in the NCG group were significantly decreased (*p* < 0.05), while, heat shock protein (*p* = 0.07) and aldosterone (*p* = 0.06) also tended to decrease. Plasma levels of C-reactive protein, cortisol, asymmetric dimethylarginine, and hypoxic-inducing factor were not significantly different (*p* > 0.10).

**Table 6:** Effect of NCG on plasma index associated with altitude stress of experimental Jersey cattle.

**Items**	**Treatments^a^**		**SEM^b^**	***P***
	**CON**	**NCG**		
C-Reactive protein (×10^3^ ng/ml)	2.99	2.68	0.40	0.72
Transferrin (mg/ml)	5.33	3.20	0.83	0.03
Heat shock proteins (ng/ml)	3.03	2.67	0.10	0.07
Endothelin (ng/L)	532	305	42.91	0.02
Thyroxine (ng/ml)	43.13	38.90	0.92	0.02
Aldosterone (ng/L)	661	493	43.89	0.06
Cortisol (ng/ml)	62	53	7.32	0.56
Asymmetric dimethylarginine (μmol/ml)	67.20	70.22	1.52	0.34
Hypoxia-inducible factor (ng/L)	303	283	7.06	0.15

a *CON, CON group, N = 6, basal diet without supplementation of N-carbamylglutamate; NCG, N = 8, treatment that added 20 (g/d/head) N-carbamylglutamate*.

b *SEM was standard error of means*.

### Metabolite Profiles of Plasma Samples

**Figure 2:**
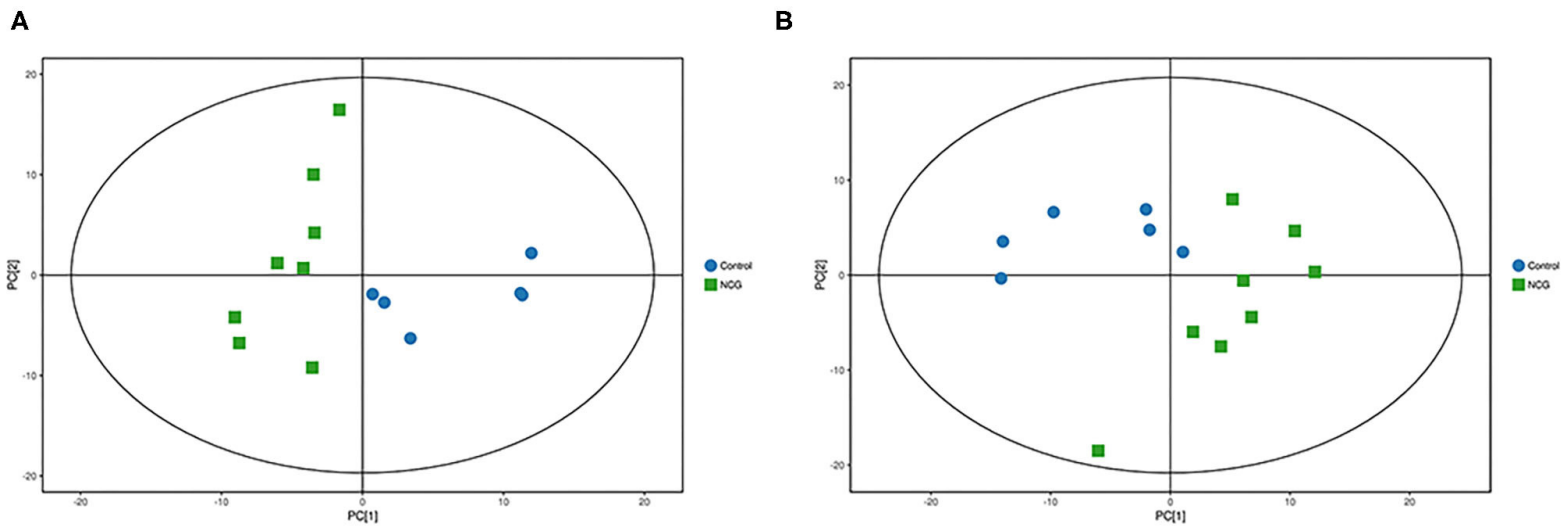
**(A)** Principal component analysis score plot for the NCG group and CON group plasma samples analyzed in the positive ion mode. **(B)** Principal component analysis score plot for the NCG and CON group samples analyzed in the negative ion mode. The abscissa PC[1] = first principal component and the ordinate PC[2] = second principal component. The blue circle represents the CON group, and the green square represents the NCG group. The samples are all in within the 95% confidence interval (Hotelling's T-squared ellipse). The blue circle represents the diet without NCG supplementation (Control, CON group, *N* = 6), and the green box represents the experimental group with 20 (g/d·head) NCG added to the diet (NCG, NCG group, *N* = 8).

**Figure 3:**
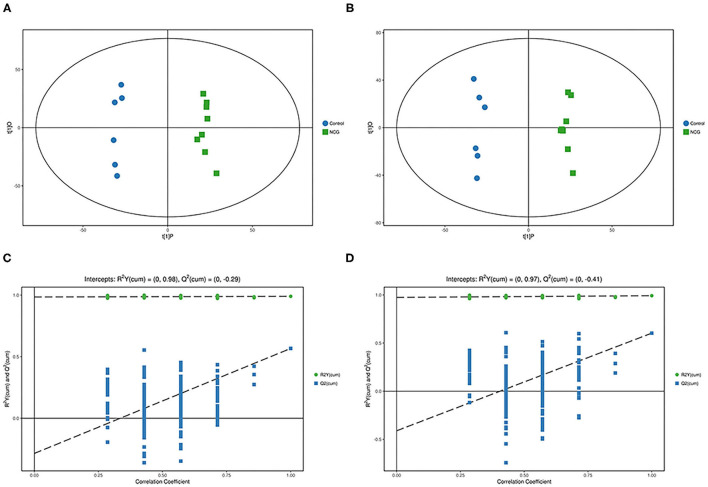
**(A)** Principal component analysis score plot for the NCG group and CON group plasma samples analyzed in the positive ion mode. **(B)** Principal component analysis score plot for the NCG and CON group samples analyzed in the negative ion mode. The abscissa PC[1] = first principal component and the ordinate PC[2] = second principal component. The blue circle represents the CON group, and the green square represents the NCG group. The samples are all in within the 95% confidence interval (Hotelling's T-squared ellipse). The blue circle represents the diet without NCG supplementation (Control, CON group, *N* = 6), and the green box represents the experimental group with 20 (g/d·head) NCG added to the diet (NCG, NCG group, *N* = 8).

**Figure 4:**
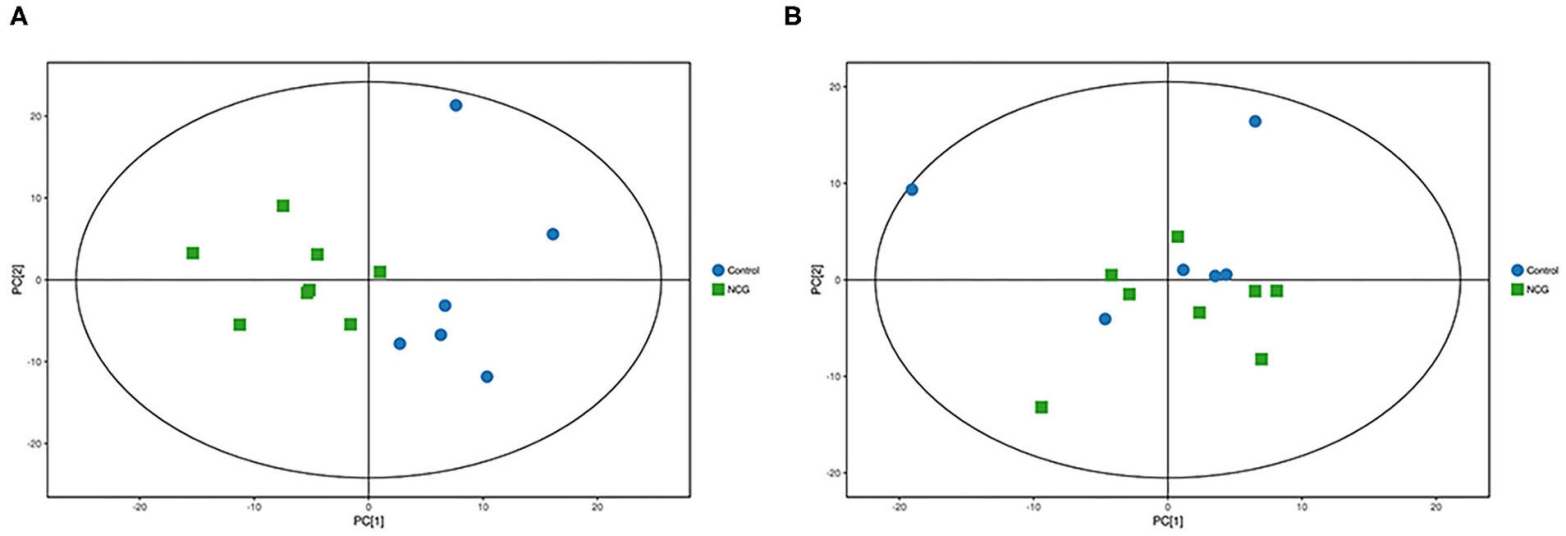
**(A)** Principal component analysis score plot for the NCG group and CON group feces samples analyzed in the positive ion mode. **(B)** Principal component analysis score plot for the NCG and CON group samples analyzed in the negative ion mode. The abscissa PC[1] = first principal component and the ordinate PC[2] = second principal component. The blue circle represents the CON group, and the green square represents the NCG group. The samples are all in within the 95% confidence interval (Hotelling's T-squared ellipse). The blue circle represents the diet without NCG supplementation (Control, CON group, *N* = 6), and the green box represents the experimental group with 20 (g/d/head) NCG added to the diet (NCG, NCG group, *N* = 8).

**Figure 5:**
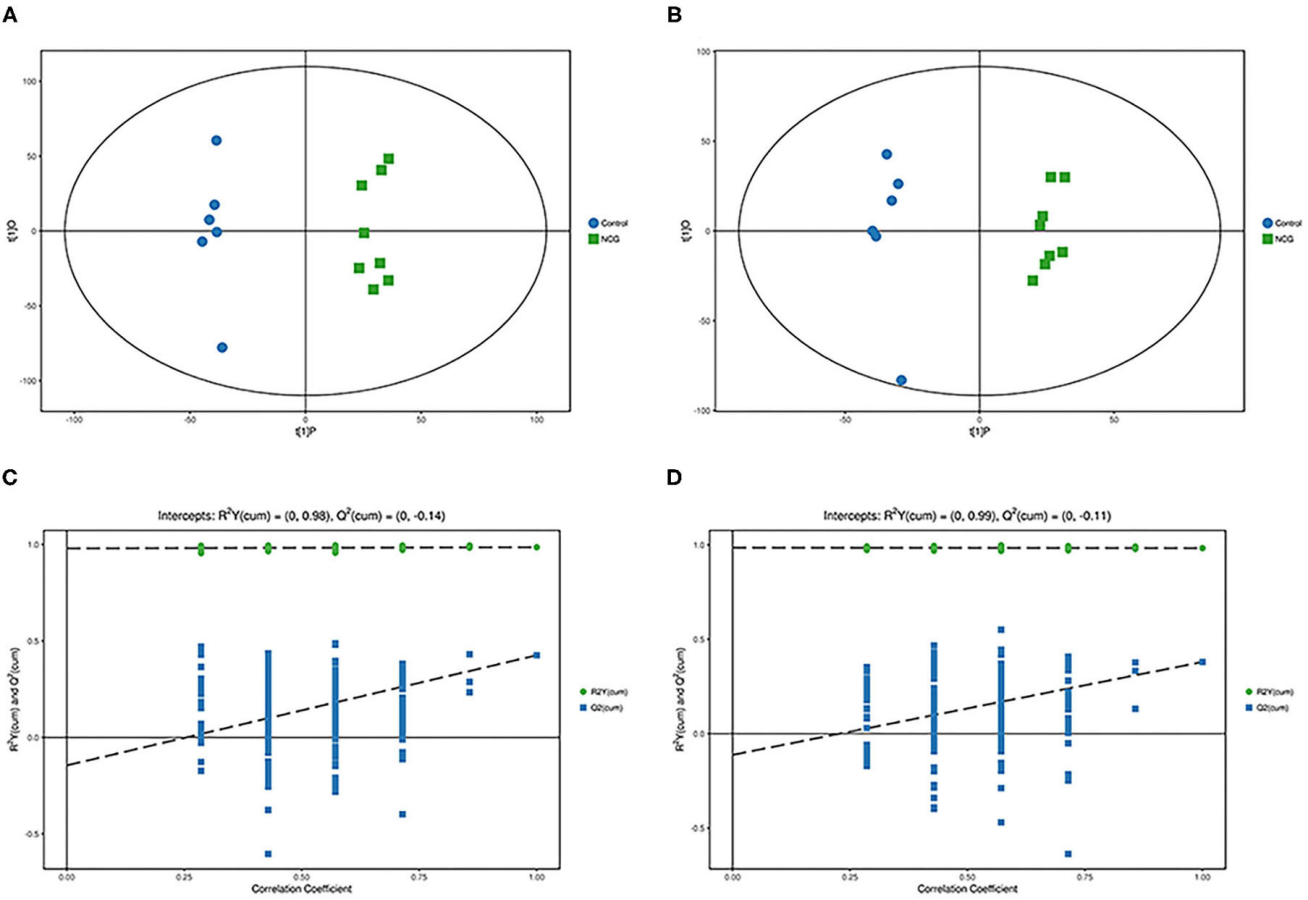
**(A,C)** Orthogonal partial least square–discriminant analysis of scores and permutation test plots for the NCG group and CON group feces samples analyzed in the positive ion mode, respectively. **(B,D)** Orthogonal partial least square–discriminant analysis of scores and permutation test plots for the NCG and CON group feces samples analyzed in the negative ion mode, respectively. t[1]P = first principal component score. t[1]O = orthogonal principal component score. The intercept limit of *Q*^2^, calculated by regression line, is the plot of *Q*^2^ from permutation test in the OPLS–DA model. The blue circle represents the diet without NCG supplementation (Control, CON group, *N* = 6), and the green box represents the experimental group with 20 (g/d/head) NCG added to the diet (NCG, NCG group, *N* = 8).

For plasma and feces samples, the PCA score plot showed that the model interpretation rates for the NCG and CON groups under the positive and negative ion mode conditions were *R*^2^*X* = 0.548 and 0.506, *R*^2^*X* = 0.506 and 0.565, respectively. Except for stool samples in negative ion mode (Figure 3B), other samples were well separated, and samples in the same group were well aggregated together (Figures 2A,B, 3A). An OPLS-DA supervised model was used to assess inter-group sample differences. During the analysis and fitting process of plasma and feces samples, in the positive ion mode of the OPLS–DA score plot, *R*^2^*Y* = 0.99 and *Q*^2^ = 0.567, *R*^2^*Y* = 0.992 and *Q*^2^ = 0.603, respectively, whereas in the negative ion mode, *R*^2^*Y* = 0.992 and *Q*^2^ = 0.603, *R*^2^*Y* = 0.983 and *Q*^2^ = 0.380, respectively. From the results of OPLS–DA score, whether for serum samples or stool samples, that can be seen that the two sets of samples were distinguished significantly, and the samples were all within the 95% confidence interval (Hotelling's T-squared ellipse), which indicating the model was stable and reliable (Figures 4A,B, 5A,B). The OPLS–DA model *R*^2^*Y* was ~1, indicated that the established model conforms to the real situation of the sample data, and *Q*^2^ is greater than 0.4, which indicated that the OPLS–DA model could better explain the difference between the two sets of samples and there was no over-fitting (Figures 4C,D, 5C,D).

### Differential Metabolite Analysis

**Figure 6:**
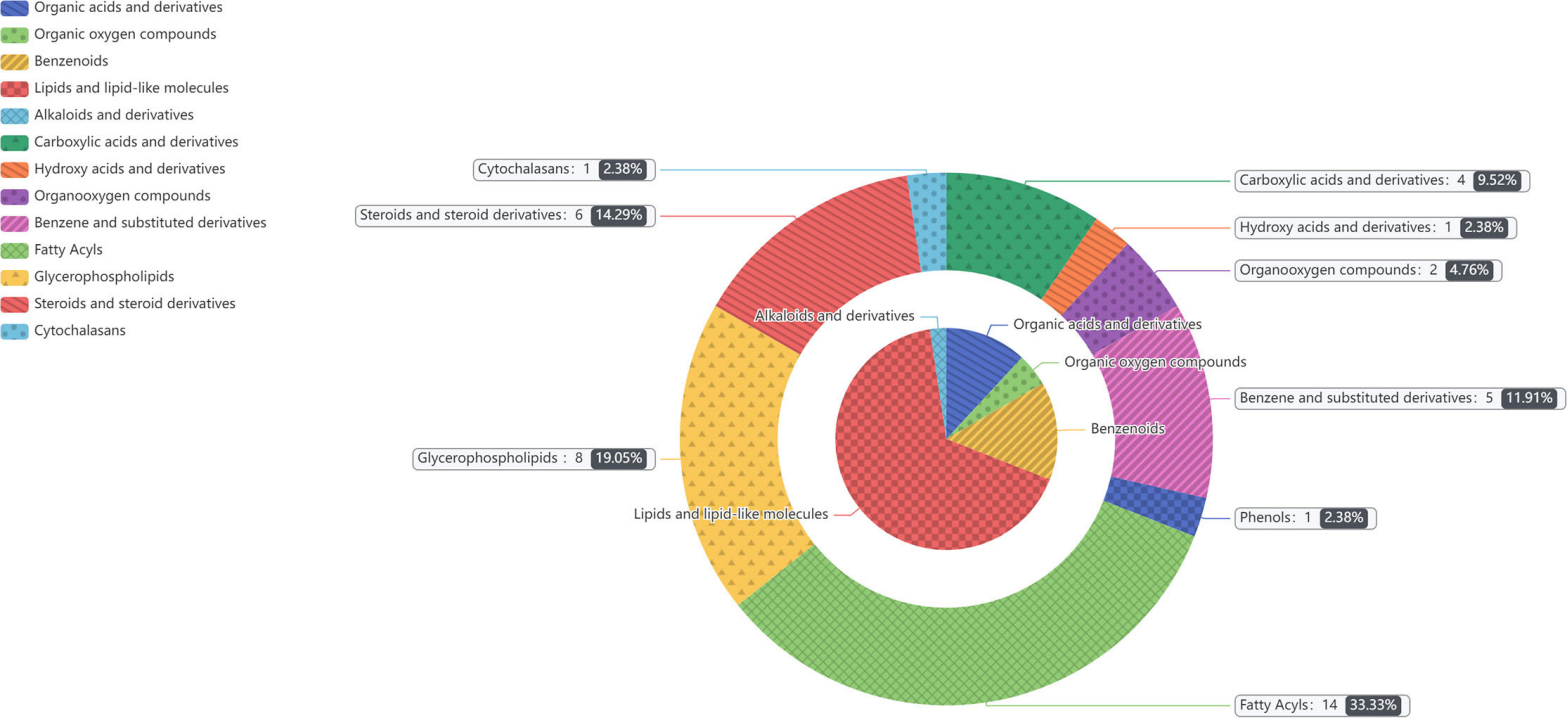
The different metabolites in plasma between NCG group (*N* = 8) and CON group (*N* = 6) are classified according to the HMDB Compound Classification. The inner pie chart is the classification of the differential metabolites in the Super Class, and the outer pie chart is the classification of the differential metabolites in the Class, and the Class belongs to the Super Class. The contents of gray box corresponding to the outer pie guided by the indicator line represents the name of the Class, the number and proportion of differential metabolites in the plasma.

**Figure 7:**
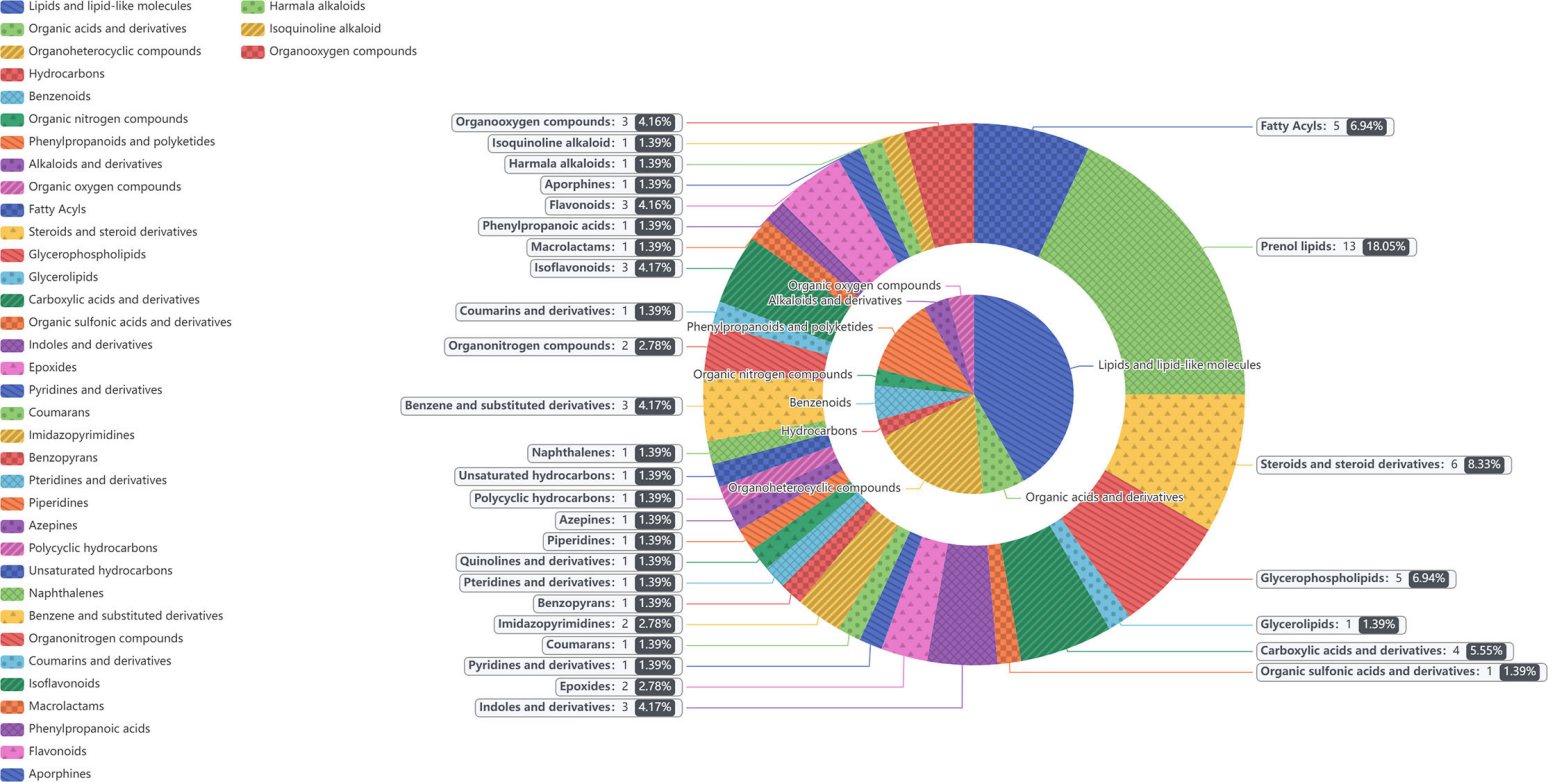
The different metabolites in feces between NCG group (*N* = 8) and CON group (*N* = 6) are classified according to the HMDB Compound Classification. The inner pie chart is the classification of the differential metabolites in the Super Class, and the outer pie chart is the classification of the differential metabolites in the Class, and the Class belongs to the Super Class. The contents of gray box corresponding to the outer pie guided by the indicator line represents the name of the Class, the number and proportion of differential metabolites in the feces.

In the two samples of plasma and feces, with *p* < 0.05 and the VIP of the first principal component of OPLS–DA model greater than 1 as the critical value, and we identified a total of 114 differential metabolites. Among them, 42 metabolites were identified in plasma samples and 72 metabolites were identified in feces. Compound classification of differential metabolites in the plasma and feces samples were processed based on comparisons with the HMDB. Differential metabolites from plasma can be divided into 5 categories at the Super Class level, mainly including lipids and lipid-like molecules, benzenoids, organic acids, derivatives, etc. (Table 7), among which lipids and lipid-like molecules have the largest number of differential metabolites, which was 28. Compared to the CON group, the levels of differential metabolites classified as benzenoids in the NCG group, all increased significantly in plasma. The significantly increased plasma differential metabolites were 2,5-dihydroxybenzoate and salicyluric acid (fold change > 2). The detailed division of each Super Class can be divided into Class, and the number and proportion of plasma differential metabolites distributed in each Class are shown in Figure 6, lipids and lipid-like molecules mainly include fatty acyls, glycerophospholipids, steroids, and steroid derivatives. In addition, organic acids and derivatives and benzenoids are mainly based on carboxylic acids and derivatives and benzene and substituted derivatives, respectively. The differential metabolites identified from feces samples are more abundant, which can be divided into 9 types in the Super Class level (Table 8), and the significantly increased fecal differential metabolites were Butenafine (fold change > 2). Consistent with the results identified in plasma samples, lipids and lipid-like molecules also accounted for the largest percentage of differential metabolites identified in feces, with a total of 30 identified. Furthermore, there are two types of organoheterocyclic compounds and phenylpropanoids and polyketides also account for a large proportion, which have been identified 15 and 9 different metabolites, respectively. Similarly, the differential metabolites identified in the feces were divided into Class on the basis of Super Class, and the corresponding number and proportion of differential metabolites are shown in Figure 7. Different from the plasma differential metabolites, the differential metabolites from feces accounted for the largest proportion of lipids and lipid-like molecules in the classification of lipids and lipid-like molecules is prenol lipids. Different from plasma differential metabolites, in feces differential metabolites that prenol lipids occupy the largest proportion in lipids and lipoid-like molecules.

**Table 7:** HMDB compound classification of differential metabolites of plasma between NCG group and CON group^a^.

**Super class^b^**	**Metabolite^c^**	***RT* (s)^d^**	**m/z^e^**	**VIP^f^**	***p*-value^g^**	**Fold change^h^**
Alkaloids and derivatives	Cytochalasin B	278.052	480.276	2.343	0.015	0.171
Benzenoids	Hippuric acid	200.125	180.065	1.846	0.014	1.456
	4S,5S-antillatoxin A	436.075	504.343	2.310	0.001	1.390
	2,5-Dihydroxybenzoate	184.632	153.019	2.346	0.001	2.028
	Salicyluric acid	165.388	194.046	2.244	0.002	2.026
	3-Hhydroxybenzoate	155.842	137.024	2.192	0.002	1.623
	Catechol	148.222	109.029	1.647	0.039	1.390
Lipids and lipid-like molecules	PGF1 alpha	419.822	325.273	1.784	0.017	1.416
	PC (7:0/O-8:0)	393.329	482.323	1.453	0.047	1.257
	PE (18:0/0:0)	463.252	482.323	1.569	0.032	0.847
	PC (P-17:0/0:0)	461.479	494.359	1.893	0.030	0.841
	LysoPC [22:5(4Z,7Z,10Z,13Z,16Z)]	416.058	570.354	1.689	0.037	0.789
	LysoPE [0:0/22:5(4Z,7Z,10Z,13Z,16Z)]	410.399	528.307	2.193	0.002	0.722
	Epigallocatechin 3-*O*-caffeate	309.076	469.109	1.989	0.005	0.647
	Glycocholic acid	309.618	466.315	1.969	0.004	0.451
	Glycodeoxycholic acid	354.305	450.320	2.085	0.002	0.443
	Smenospongiarine	350.745	414.299	2.100	0.036	0.432
	Tetranor-PGFM	224.334	329.160	1.781	0.011	1.649
	Dodecanedioic acid	193.524	229.144	1.824	0.020	1.447
	Tetradecanedioic acid	227.044	257.176	1.941	0.008	1.367
	PE [18:1(9Z)/0:0]	426.305	478.293	1.730	0.025	1.196
	Phosphatidylcholine lyso 18:1	432.200	580.361	1.911	0.008	1.162
	14-methyl palmitic acid	518.448	269.248	1.531	0.036	0.848
	Stearic acid	546.263	283.264	1.892	0.005	0.804
	Eicosa-5Z,8Z-dienoic acid [20:2, n-12]	517.388	307.264	1.957	0.004	0.803
	Phosphatidylcholine lyso 20:4	404.111	602.346	1.969	0.006	0.781
	Arachidonic Acid	463.287	303.232	1.723	0.024	0.758
	16-hydroxy hexadecanoic acid	348.369	271.228	1.993	0.004	0.729
	7Z, 10Z, 13Z, 16Z, 19Z-docosapentaenoic acid	476.463	329.248	1.917	0.007	0.726
	Stearic acid ethyl ester	613.921	311.295	1.824	0.011	0.717
	cis-9,10-Epoxystearic acid	361.554	297.243	2.073	0.004	0.656
	Docosahexanoic acid	450.270	327.232	1.841	0.032	0.638
	Glycocholate	261.714	464.301	1.799	0.004	0.466
	Tauroursodeoxycholic acid	292.435	498.289	2.390	0.011	0.244
	Taurocholic acid	266.388	514.284	2.358	0.014	0.190
Organic acids and derivatives	Pyroglutamic acid	60.847	130.050	1.676	0.043	1.943
	*N, N*-Dimethylglycine	35.107	104.070	1.836	0.020	1.451
	*L*-Ornithine	27.910	133.097	1.515	0.031	0.765
	Sarcosine	34.145	88.040	1.876	0.010	1.136
	(*R*)-2-Hydroxystearic acid	415.344	299.259	1.731	0.022	0.794
Organic oxygen compounds	2,8-Dihydroxyquinoline-beta-D-glucuronide	188.995	338.086	1.766	0.029	1.631
	Pantothenic acid	158.242	220.117	1.623	0.036	0.756

*^a^CON, CON group, N = 6, basal diet without supplementation of N-carbamylglutamate; NCG, N = 8, treatment that added 20 (g/d·head) N-carbamylglutamate*.

b *Based on the chemical structure of the metabolite in HMDB*.

c *The name of the substance that matches in the secondary mass spectrum*.

d *Retention time*.

e *Mass-to-charge ratio*.

f *ariable Importance in the Projection value from OPLS–DA model*.

g *p-Value: p-value from t-test*.

h *The quantitative ratio of the two experimental substances in the NCG group and the CON group*.

**Table 8:** HMDB compound classification of differential metabolites of feces between NCG group and CON group^a^.

**Super class^b^**	**Metabolite^c^**	***RT* (s)^d^**	**m/z^e^**	**VIP^f^**	***p*-Value^g^**	**Fold change^h^**
Alkaloids and derivatives	Lyconnotine	238.953	292.190	1.634	0.040	0.880
	Flazine	279.120	309.086	1.966	0.016	0.836
	Rodiasine	253.376	623.306	2.359	0.001	0.521
Benzenoids	Butenafine	424.995	318.219	1.929	0.025	2.550
	2-Phenylethylamine	168.051	122.096	1.723	0.041	0.662
	Styrene	168.051	105.070	1.717	0.042	0.659
	2-Iodophenol methyl ether	26.382	234.961	1.767	0.033	0.833
Hydrocarbons	5-propylideneisolongifolane	503.255	247.242	2.032	0.011	1.080
	Santene	480.754	123.117	1.855	0.024	1.115
Lipids and lipid-like molecules	Docosahexaenoic Acid ethyl ester	448.512	357.278	1.874	0.029	1.279
	Dihydroceramide C2	471.910	344.315	1.708	0.029	1.170
	Digeranyl	538.957	275.273	1.719	0.049	1.118
	4,8 dimethylnonanoyl carnitine	232.523	330.263	2.705	0.000	1.655
	Tocotrienol	489.569	411.325	1.932	0.010	0.871
	(22E, 24x)-Ergosta-4,6,8,22-tetraen-3-one	489.569	393.314	1.870	0.014	0.871
	13-Methyl-4,4-Bisnor-8,11,13-Podocarpatrien-3-ONE	489.570	229.158	1.929	0.011	0.861
	5,12-Octadecadiynoic acid	414.071	277.216	1.987	0.018	0.831
	PE (18:1(9Z)/0:0)	418.124	480.307	1.888	0.018	0.820
	Camelledionol	450.066	441.335	1.751	0.037	0.814
	ent-6,16-Kauradien-19-oic acid	474.300	301.216	1.706	0.026	0.798
	3-Oxo-5-chol-8-en-24-oic Acid	383.525	373.273	1.651	0.045	0.834
	LysoPE(0:0/18:0)	462.739	482.323	1.758	0.048	0.836
	Ruscogenin	486.895	431.315	2.091	0.005	0.430
	(2E,6E,10R,11S)-10,11-epoxy-3,7,11-trimethyltrideca-2,6-dienoic acid	306.946	267.195	1.657	0.040	0.855
	Juvenile hormone I	414.071	295.226	2.194	0.019	0.820
	3beta-Hydroxylanostane-7,11-dione acetate	402.001	501.390	2.256	0.002	0.711
	Xanthophyll	587.571	568.426	1.646	0.032	0.836
	(24R)-1,24-dihydroxy-26,27-dimethyl-22-oxavitamin D3	533.250	447.346	2.091	0.009	0.818
	30:5(15Z,18Z,21Z,24Z,27Z)	402.001	443.387	2.062	0.008	0.748
	Stenocereol	515.232	415.356	1.657	0.050	0.833
	Cassaine	379.488	406.294	2.284	0.001	0.589
	Erythroxanthin	471.623	599.417	2.114	0.006	0.691
	(11Z)-8,18-propano-retinal	379.490	325.252	2.374	0.001	0.587
	LysoPE[0:0/20:4(5Z,8Z,11Z,14Z)]	426.975	502.289	2.005	0.010	0.781
	LPA (0:0/16:0)	379.481	411.250	2.296	0.002	0.484
	25-Hydroxycholecalciferol (25-hydroxyvitamin D3)	385.926	423.325	1.876	0.017	0.811
	Glycyrrhetinic Acid	372.918	469.331	1.915	0.021	0.696
	PE (18:1(9Z)/0:0)	425.902	478.293	2.269	0.005	0.781
	13,14-dihydro-15-keto-tetranor PGF1	194.510	299.186	2.256	0.004	0.833
Organic acids and derivatives	Stearamide	534.593	284.294	2.233	0.003	1.160
	Capsi-amide	515.367	270.279	1.718	0.035	1.120
	Stearidonoyl glycine	282.881	334.237	1.629	0.041	0.898
	D-Alanine	30.044	88.040	1.783	0.037	0.844
	Sulfoacetic acid	25.733	138.970	2.057	0.008	0.673
Organic nitrogen compounds	3-Methylbutanamine	141.395	88.112	1.989	0.016	0.605
	Oleoyl ethyl amide	539.017	310.310	2.042	0.009	1.135
Organic oxygen compounds	4-Hexen-3-one	183.964	99.080	1.795	0.035	0.464
	Adlupone	490.932	483.343	1.947	0.015	0.730
	D-Arabinono-1,4-lactone	27.111	147.030	2.058	0.010	0.694
Organoheterocyclic compounds	3-Indoleacetic acid	255.861	176.070	1.684	0.036	1.422
	6R,7S-Epoxy-3Z,9Z-eicosadiene	539.007	293.283	2.104	0.008	1.141
	9S,10R-Epoxy-6Z-octadecene	503.254	265.252	1.965	0.014	1.065
	4-Hydroxyindole	224.457	134.060	1.721	0.027	0.724
	3-Formyl-6-hydroxyindole	223.695	162.055	2.134	0.020	0.787
	Nicotinamide	168.049	123.100	1.707	0.043	0.655
	Anisoxide	489.569	203.143	1.853	0.013	0.863
	Guanine	135.625	152.057	1.809	0.027	0.830
	6-Deoxyjacareubin	236.797	311.091	1.648	0.036	0.847
	2,3-dihydro-2-oxo-1H-Benzimidazole-1-propanoic acid	174.236	207.076	1.723	0.029	0.867
	Aurachin D	488.775	364.263	1.493	0.041	0.807
	Dihydrozeatin	43.971	222.133	1.718	0.022	0.749
	Loxtidine	241.352	360.237	1.987	0.013	0.707
	2-Acetyl-1,5,6,7-tetrahydro-6-hydroxy-7-(hydroxymethyl)-4H-azepine-4-one	153.616	200.092	1.556	0.041	0.846
Phenylpropanoids and polyketides	4-methylumbelliferone	355.658	177.054	1.484	0.046	1.076
	6-(1,1-Dimethylallyl) genistein	380.028	339.122	2.239	0.003	0.845
	Neodunol	238.958	281.080	1.980	0.007	0.827
	Geldanamycin Analog	468.118	568.266	2.040	0.028	0.776
	3-Phenylpropionic acid	181.294	149.061	1.635	0.042	0.822
	8-Prenylapigenin	378.535	337.108	2.052	0.020	0.880
	8-Prenylnaringenin	365.431	339.123	1.740	0.046	0.665
	7,4'-Dimdthoxyisoflavone	169.720	281.081	1.865	0.014	0.834
	6-Geranylnaringenin	415.770	407.186	2.162	0.011	0.899

*^a^CON, CON group, N = 6, basal diet without supplementation of N-carbamylglutamate; NCG, N = 8, treatment that added 20 (g/d·head) N-carbamylglutamate*.

b *Based on the chemical structure of the metabolite in HMDB*.

c *The name of the substance that matches in the secondary mass spectrum*.

d *Retention time*.

e *Mass-to-charge ratio*.

f *Variable Importance in the Projection value from OPLS–DA model*.

g *P-value: P value from t-test*.

h *The quantitative ratio of the two experimental substances in the NCG group and the CON group*.

**Figure 8:**
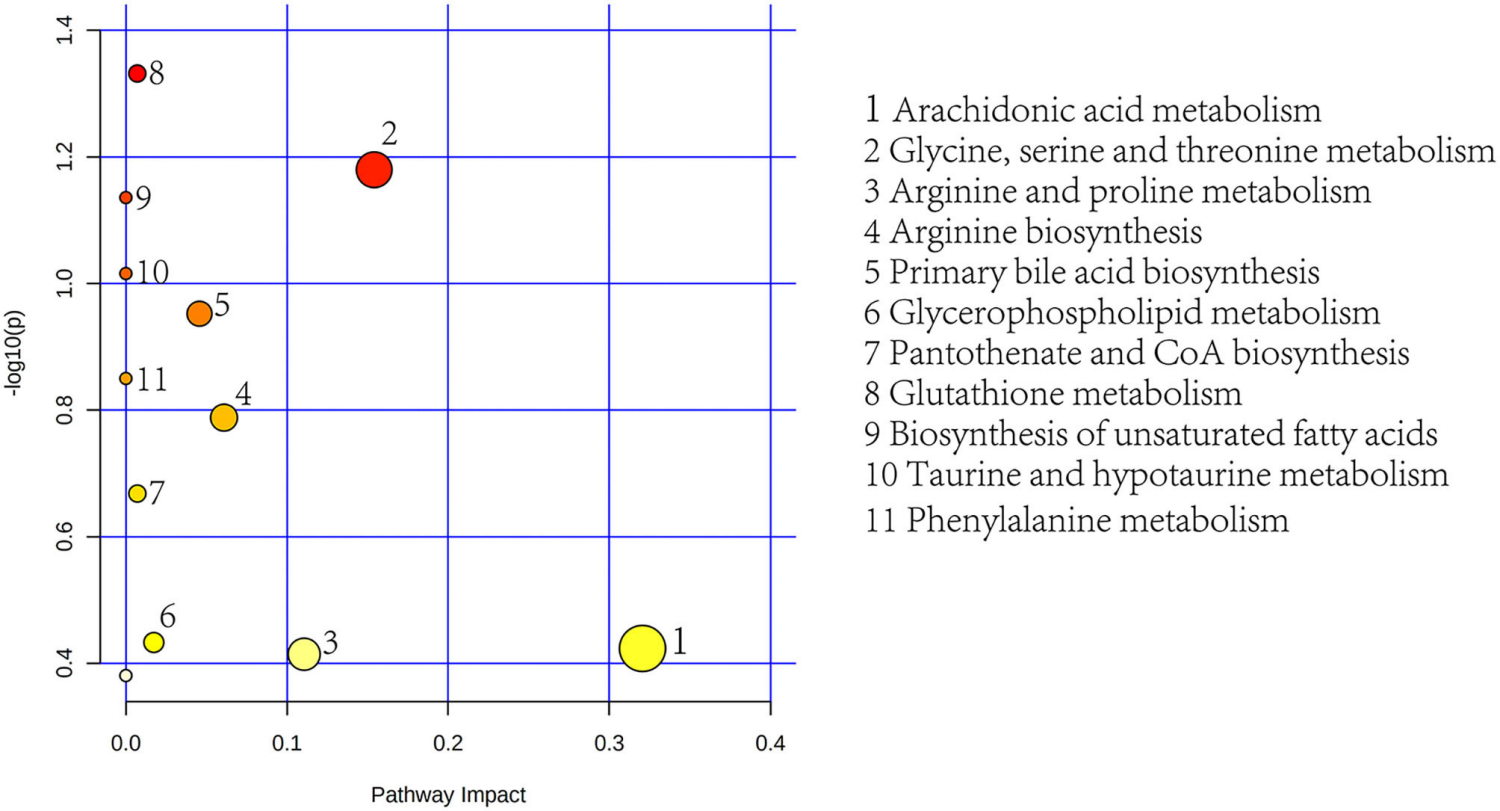
Plasma metabolic pathway analysis using MetaboAnalyst 5.0 (http://www.metaboanalyst.ca; *x*-axis, pathway impact; *y*-axis, –log*p*). Circles represent metabolic pathways. Darker circles indicate more significant changes in the metabolites in the corresponding pathway, whereas the size of the circle corresponds to the pathway impact score.

**Figure 9:**
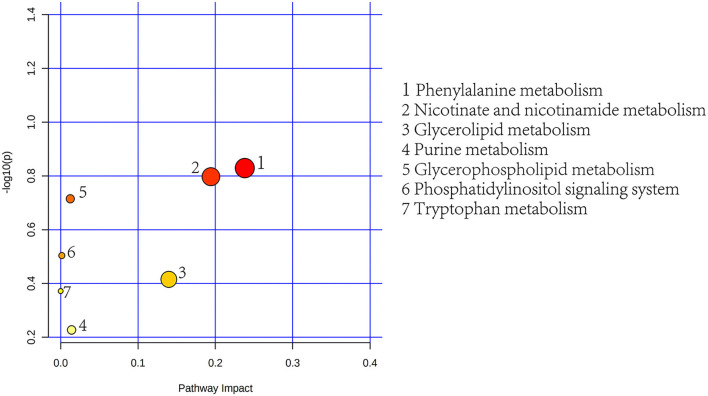
Feces metabolic pathway analysis using MetaboAnalyst 5.0 (http://www.metaboanalyst.ca). *x*-Axis, pathway impact; *y*-axis, –log(*p*). Circles represent metabolic pathways. Darker circles indicate more significant changes in the metabolites in the corresponding pathway, whereas the size of the circle corresponds to the pathway impact score.

To further determine the biological significance of the differential metabolites, we searched differential metabolites in the KEGG and performed a metabolic pathway analysis using MetaboAnalyst 5.0 (https://dev.metaboanalyst.ca). After drawing into a bubble chart, we found that plasma differential metabolites can be enriched in a total of 11 metabolic pathways (Figure 8), mainly lipid metabolism and amino acid metabolism. These include Arg and proline metabolism and Arg biosynthesis, two metabolic pathways directly related to Arg synthesis. Similarly, the differential metabolites in feces were enriched in 7 metabolic pathways, mainly aromatic amino acid metabolism and lipid metabolism pathways (Figure 9). In addition, differential metabolites from plasma and feces can be enriched to two metabolic pathways in common, which are phenylalanine metabolism and glycerophospholipid metabolism.

## Discussion

Compared with the additive dosage of 20 g/d/head NCG that we referred to and used is suitable for Holstein cattle in low-altitude areas (22), and our results of the same dosage on the plateau showed that there is a certain effect trend in improving blood oxygen saturation and affecting the index associated with altitude stress, which is directly related to symptoms such as HAH and HAPH. Previous studies by Wang et al. (27) have shown that lower blood oxygen saturation levels are independent risk factors for HAH. At the same time, HAPH can make blood oxygen saturation much lower than health level (28). Meanwhile, the addition of NCG can be seen from the Index associated with altitude stress and still plays a regulatory role. Among them, endothelin plays an important role in maintaining basal vascular tension and cardiovascular system homeostasis (29), and it is one of the strongest vasoconstrictors found so far. It participates in the formation of pulmonary hypertension and inhibits the blood supply of microvascular blood in the brain tissue microvascular (30, 31). Besides, the large amount of thyroxine secretion under hypoxia stimulation will cause excessive metabolism consumption of the body (32). However, the contents of transferrin, endothelin and thyroxine in the NCG group decreased significantly, which can be speculated that NCG can inhibit the formation of pulmonary hypertension and reduce body consumption by reducing the secretion of transferrin, endothelin and thyroxine to some extent, thereby further alleviating HAH and HAPH. In addition, under the condition of high-altitude hypoxia environment, the contents of molecules that are positively correlated with hypoxia stress response, such as heat shock protein and aldosterone, also have a decreased trend in the NCG group. These data prove that NCG has a certain mitigation effect on HAH and HAPH.

For milk quality test results, there is almost no difference in milk protein, but milk fat shows an upward trend, which is consistent with the research by (33). Interestingly, in the analysis results of plasma non-targeted metabolomics, we found that under the confidence condition of *p* < 0.05, the mass spectrometry value of the metabolite hippuric acid in the plasma of NCG group was significantly higher than that of the CON group with a fold change of 1.456. In the previous studies by (34), it was shown that hippuric acid is a marker metabolite for measuring milk production in high-yield lactating cow, and there are reports that hippuric acid has a positive correlation with milk yield and quality can effectively reflect the level of milk production in dairy cows, and the higher serum hippuric acid levels might also indicate more energy supplied by glucose metabolism and hormone regulation (35). Due to the limitation of experimental conditions, we could not measure the data of milk yield, but the existing results still suggest that the addition of NCG has a certain effect on milk quality for dairy cows reared in the plateau environment. These results all indicate that the NCG concentration suitable for high altitude is still worthy of further investigation, compared with the NCG supplemental level at low altitude.

Through the classification of 114 different metabolites in plasma and feces, it was found that lipids and lipid-like molecules accounted for the largest proportion in the two samples. The analysis of the metabolic pathways involved in these differential metabolites found that whether it is plasma samples or stool samples, they are mainly related to amino acid metabolism and lipid metabolism, such as glycerophospholipid metabolism as the common impact pathway identified in plasma and feces, which is directly related to milk fat synthesis. Meanwhile, among the plasma metabolites, two metabolic pathways are directly related to Arg, including Arg and proline metabolism and Arg biosynthesis. Combined with the previously mentioned effects of Arg on vascular tension, blood flow and blood pressure (15), it is also shown here that the addition of NCG in the diet may have a certain effect on the synthesis of Arg in the dairy cow, which is consistent with previous studies (5, 36). Whether the effect of NCG on Arg metabolism is related to the previous result that blood oxygen saturation has a tendency to increase is worthy of further study. At the same time, we noticed that the metabolic pathway of plasma with the greatest influence is arachidonic acid metabolism, and arachidonic acid is usually considered to be a precursor to a number of potent pro-inflammatory mediators (37), and is positively correlated with fat accumulation in the liver (38). However, arachidonic acid as a metabolite that is significantly reduced in the plasma samples. In addition, the fold change of 3-indoleacetic, which is the difference metabolite detected in feces between the NCG group and CON, reached 1.422, and previous studies have shown that it can improve plasma T-AOC and inhibit the decrease of liver SOD activity, thereby reduced the oxidative and inflammatory stress of liver tissue (39). Whether the above evidence suggests that NCG can also be effective against inflammation requires further experimental verification.

## Conclusions

In conclusion, our results indicate that dietary addition of NCG with 20 (g/d/head) can regulate index associated with altitude stress such as thyroxine, transferrin, and endothelin in lactating Jersey cattle at high altitude, and is conducive to the improvement of blood oxygen saturation, thus alleviating the hypoxia stress state of Jersey cattle. The addition of NCG does not affect the nutrient digestion and immunity, but it tends to increase milk fat and affects the rumen fermentation parameters and causes butyric acid to decrease tendency. Analysis of 114 differential metabolites identified from plasma samples and feces samples showed that these changes were mainly reflected in amino acid metabolism and lipid metabolism. These data suggest that NCG is still beneficial to the improvement of physiological state and production performance of Jersey cattle at high-altitude areas, which provide theoretical basis for NCG to prevent altitude sickness and regulate nutritional physiology of dairy cows. However, the additive dosage needs to further consider the impact of environmental factors and interspecies differences.

## Data Availability Statement

The raw data supporting the conclusions of this article will be made available by the authors, without undue reservation.

## Ethics Statement

The animal study was reviewed and approved by Animal Care and Use Guidelines of the Animal Care Committee, Institute of Subtropical Agriculture, Chinese Academy of Sciences, Changsha, China (Approval Number: ISA-EA2019-7).

## Author Contributions

CZ, TJ, and ZT: conceived and designed the experiments. ZL, HM, XL, KW, and XH: performed the experiments. ZL, BL, LC, and GZ: analyzed the data. ZL, CZ, and FY: contributed to the writing of the manuscript. All authors reviewed and approved the manuscript.

## Funding

This work was jointly supported by the Special Item of Regional Collaborative Innovation in Tibet Autonomous Region and Science (QYXTZX-LS2021-01), National Natural Science Foundation of China (31730092), Strategic Priority Research Program of Chinese Academy of Sciences (XDA26040306), Hunan Provincial Science and Technology Department Project (2020WK4002), and Technology Department of Tibet (XZ202101ZD003N).

## Conflict of Interest

FY was employed by Hunan Jiuding Technology (Group) Co., Ltd. XH was employed by Changsha Green Top Biotech Co., Ltd. The remaining authors declare that the research was conducted in the absence of any commercial or financial relationships that could be construed as a potential conflict of interest.

## Publisher's Note

All claims expressed in this article are solely those of the authors and do not necessarily represent those of their affiliated organizations, or those of the publisher, the editors and the reviewers. Any product that may be evaluated in this article, or claim that may be made by its manufacturer, is not guaranteed or endorsed by the publisher.

## Abbreviations

ADF: acid detergent fiber
AIA: acid insoluble ash
AOAC: Official analytical chemists methodologies
Arg: arginine
ATTD: apparent total tract digestibility
Ca: calcium
CAT: catalase
CON: control
CP: crude protein
CPS-I: carbamoyl phosphate synthase-I
DM: dry matter
DMI: dry matter intake
EDTA: ethylene diamine tetraacetic acid
ELISA: enzyme linked immunosorbent assay
GE: gross energy
HAH: high-altitude hypoxia
HAPH: high-altitude pulmonary hypertension
HMDB: human metabolome database
IgA: immunoglobulin A
IgG: immunoglobulin G
IgM: immunoglobulin M
KEGG: Kyoto Encyclopedia Genes and Genomes pathway database
LC–MS: liquid chromatography–mass spectrometry
MDA: malondialdehyde
NCG: *N*-carbamoylglutamate
NDF: neutral detergent fiber
NH_3_-N: ammonia nitrogen
NOS: nitric oxide synthase
OM: organic matter
OPLS–DA: orthogonal partial least squares–discriminate analysis
P: phosphorus
PCA: principle component analysis
RT: retention time
SEM: standard error of means
SOD: superoxide dismutase
T-AOC: total antioxidant capacity
TMR: total mixed rations
VFAs: volatile fatty acids
VIP: variable importance in the projection.
